# Classifiers Combined with DenseNet Models for Lung Cancer Computed Tomography Image Classification: A Comparative Analysis

**DOI:** 10.2174/0115734056399377250818100506

**Published:** 2025-08-26

**Authors:** Menna Allah Mahmoud, Sijun Wu, Ruihua Su, Yanhua Wen, Shuya Liu, Yubao Guan

**Affiliations:** 1 Department of Radiology, The Fifth Affiliated Hospital of Guangzhou Medical University, Guangzhou, China

**Keywords:** Lung cancer, CT scans, Deep learning, Medical imaging, SVM, Feature extraction method, Data augmentation, MLP

## Abstract

**Introduction:**

Lung cancer remains a leading cause of cancer-related mortality worldwide. While deep learning approaches show promise in medical imaging, comprehensive comparisons of classifier combinations with DenseNet architectures for lung cancer classification are limited.

The study investigates the performance of different classifier combinations, Support Vector Machine (SVM), Artificial Neural Network (ANN), and Multi-Layer Perceptron (MLP), with DenseNet architectures for lung cancer classification using chest CT scan images.

**Methods:**

A comparative analysis was conducted on 1,000 chest CT scan images comprising Adenocarcinoma, Large Cell Carcinoma, Squamous Cell Carcinoma, and normal tissue samples. Three DenseNet variants (DenseNet-121, DenseNet-169, DenseNet-201) were combined with three classifiers: SVM, ANN, and MLP. Performance was evaluated using accuracy, Area Under the Curve (AUC), precision, recall, specificity, and F1-score with an 80-20 train-test split.

**Results:**

The optimal model achieved 92% training accuracy and 83% test accuracy. Performance across models ranged from 81% to 92% for training accuracy and 73% to 83% for test accuracy. The most balanced combination demonstrated robust results (training: 85% accuracy, 0.99 AUC; test: 79% accuracy, 0.95 AUC) with minimal overfitting.

**Discussion:**

Deep learning approaches effectively categorize chest CT scans for lung cancer detection. The MLP-DenseNet-169 combination's 83% test accuracy represents a promising benchmark. Limitations include retrospective design and a limited sample size from a single source.

**Conclusion:**

This evaluation demonstrates the effectiveness of combining DenseNet architectures with different classifiers for lung cancer CT classification. The MLP-DenseNet-169 achieved optimal performance, while SVM-DenseNet-169 showed superior stability, providing valuable benchmarks for automated lung cancer detection systems.

## 1 INTRODUCTION

Lung cancer remains a leading cause of cancer-related mortality worldwide, underscoring the critical need for early and accurate diagnosis to improve patient outcomes [1]. Computed Tomography (CT) scans play a pivotal role in lung cancer detection and characterization, providing detailed anatomical information that aids in the identification of suspicious lesions and the assessment of disease progression [2].

However, the interpretation of CT scans can be challenging and time-consuming, even for experienced radiologists, due to the subtle variations in tumor appearance and the presence of other pulmonary abnormalities [3].

Computer-Aided Diagnosis (CAD) systems based on Artificial Intelligence (AI) have emerged as promising tools to augment radiologists' expertise and improve the efficiency and accuracy of lung cancer diagnosis [4]. In particular, deep learning, a subfield of AI that leverages artificial neural networks to learn complex patterns from data, has demonstrated remarkable success in various medical image analysis tasks, including lung nodule detection, classification, and segmentation [5].

Among the different deep learning architectures, Densely Connected Convolutional Networks (DenseNets) have garnered attention for their ability to alleviate the vanishing-gradient problem and promote feature reuse, leading to improved performance and parameter efficiency [6]. Recent advances in DenseNet architectures have shown promising results in lung cancer classification. Saied *et al*. (2023) achieved 90.39% accuracy using DenseNet-121 for pulmonary nodule classification, establishing a strong baseline for automated detection systems [7]. Building upon this foundation, Uddin *et al*. (2024) introduced an attention-enhanced DenseNet architecture that improved model interpretability while maintaining high classification performance [8]. Lanjewar *et al*. (2023) further advanced the field by implementing a modified DenseNet-201 model with sophisticated feature selection methods, achieving accuracy rates of 95-100% [9]. Additionally, Arzmi *et al*. (2023) demonstrated the effectiveness of combining DenseNet with Support Vector Machine (SVM) classifiers, reporting 87% accuracy with their DenseNet-121-SVM implementation [10].

Despite these advancements, challenges persist in developing robust and generalizable models for lung cancer classification. The choice of network architecture, classification algorithm, and feature extraction method significantly impacts model performance [11, 12]. Overfitting remains a concern, particularly with limited datasets, necessitating larger, diverse datasets and advanced validation methods [12, 13]. Additionally, the interpretability of deep learning models is crucial for clinical adoption, requiring a balance between accuracy and explainability [14].

While previous studies have demonstrated the effectiveness of DenseNet combined with Support Vector Machine (SVM) combinations for lung cancer classification [ 10], a systematic comparison of ANN and MLP classifiers with DenseNet variants remains unexplored. This comparative evaluation is clinically important, as different classifiers may offer varying trade-offs between accuracy, computational efficiency, and interpretability—factors crucial for clinical deployment of automated lung cancer detection systems.

This study aims to conduct a comprehensive evaluation of different classifier combinations (SVM, ANN, and MLP) with DenseNet architectures for classifying CT images of lung cancer, comparing their performance metrics and identifying optimal combinations for clinical applications.

## 2 MATERIALS AND METHODS

### 2.1 Study Design

This experimental study utilized deep learning classification methods for lung cancer classification from CT scan images. The study employed a comparative analysis design to evaluate multiple machine learning algorithms on a standardized dataset.

### 2.2 Dataset

This study utilized the Chest CT-Scan images dataset from Kaggle (Chest CT-Scan images dataset), comprising 1000 CT scan images across four categories: Adenocarcinoma (338 images), Large Cell Carcinoma (187 images), Squamous Cell Carcinoma (260 images), and normal cells (215 images) [15].

### 2.3 Data Preparation and Augmentation

Images were preprocessed and augmented using TensorFlow's ImageDataGenerator. Augmentation techniques included rescaling, rotation, shifting, and flipping. The target size for model input was set to 224x224 pixels.

### 2.4 Feature Extraction

Three variants of the DenseNet architecture-DenseNet-121, DenseNet-169, and DenseNet-201— were employed as feature extractors. These pre-trained models were used to generate feature maps for subsequent classification.

### 2.5 Classifier Implementation

SVM was selected based on its proven effectiveness in lung cancer classification literature [10]. ANN and MLP classifiers were chosen to provide a systematic comparison of different nonlinear approaches with varying architectural complexities and learning mechanisms. SVM with RBF kernel employs kernel-based transformations to create complex decision boundaries, while ANN and MLP utilize multi-layer neural architectures for feature learning. This comprehensive comparison across different algorithmic approaches allows identification of optimal classifier-feature extractor combinations for lung cancer detection.

### 2.6 Classification Methods and Model Architecture

The study implemented three distinct classification approaches in combination with DenseNet architectures:

- Support Vector Machine (SVM) Classification
  - Features extracted from DenseNet models were fed into SVM classifiers.
  - Radial Basis Function (RBF) kernel implementation.
  - Model regularization using the C parameter.
- Artificial Neural Network (ANN) Architecture
  - Three-stage neural network implementation.
  - First hidden layer: 512 units with ReLU activation.
  - Second hidden layer: 256 units with ReLU activation.
  - Dropout regularization (50% first layer, 30% second layer).
  - Final layer: Softmax activation for multi-class output.
  - Training configuration: Adam optimizer, categorical cross-entropy loss
- Multi-Layer Perceptron (MLP) Implementation
  - Three-layer architecture:
  - Input layer: 128 units with ReLU activation.
  - Hidden layer: 64 units with ReLU activation.
  - Output layer: 4 units (matching class count).
  - L2 regularization for weight matrices.
  - Early stopping mechanism with patience = 5.

### 2.7 Model Training and Evaluation

We employed a hold-out validation approach with an 80-20 train-test split, utilizing a fixed random state to ensure reproducibility.

The ANNs were trained for 10 epochs, using categorical cross-entropy loss and the Adam optimizer. The MLP was trained for a maximum of 50 epochs with early stopping.

Performance was assessed using accuracy, Area Under the Curve (AUC), precision, recall, and F1-score. Overall performance metrics were calculated using a weighted average to account for potential class imbalance or differences in class importance. Confusion matrices and ROC curves were also generated to evaluate model performance on individual classes.

#### 2.7.1 Gradient-weighted Class Activation Mapping (GradCAM) Implementation

Gradient-weighted Class Activation Mapping (GradCAM) was implemented to provide visual explanations of model predictions using TensorFlow/Keras libraries. GradCAM heatmaps were generated using the last convolutional layer (conv5_block32_2_conv) of DenseNet-169 to highlight regions influencing classification decisions. For the MLP model, a full end-to-end architecture was reconstructed by combining the DenseNet-169 feature extractor with the trained MLP classifier layers. For SVM visualization, a neural network approximation of the SVM decision boundaries was created to enable GradCAM analysis, as traditional SVM models are not directly compatible with gradient-based visualization techniques. A total of 40 randomly selected images (10 per class) were used for visualization analysis.

### 2.8 Hardware and Packages

This study was conducted using Python 3.9.18 within the Anaconda3 distribution on a Windows 10 Home operating system. The key packages utilized include TensorFlow 2.10.0 (with its integrated Keras library), NumPy 1.26.4, Pandas 2.2.2, Scikit-learn 1.42, Matplotlib 3.9.0, and Seaborn 0.13.2. The development environment was Jupyter Notebook. The hardware configuration consisted of an Intel Core i7 (7th generation) processor, 16 GB of RAM, and an NVIDIA GeForce GTX 1080 Ti GPU for accelerated computations.

## 3 RESULTS

The integration of deep learning architectures with traditional classifiers presents promising opportunities for medical image analysis. This study systematically evaluated multiple combinations of neural network architectures and classification methods for chest CT scan analysis.

The evaluation metrics across all models demonstrated consistent patterns in both training and testing phases. For the training set, accuracy ranged from 81% to 92% with Area Under the Curve (AUC) values between 0.96 and 0.99. Test set performance showed accuracy ranges from 73% to 83% with AUC values between 0.92 and 0.95.

Among all tested combinations, the Multi-Layer Perceptron (MLP) classifier combined with DenseNet-169 emerged as the highest-performing model, achieving 92% accuracy on the training set and 83% accuracy on the test set. Fig. (**1**) illustrates this performance through ROC curves for both training (A) and test (B) sets, while Fig. (**2**) presents the corresponding confusion matrices, providing detailed insight into the model's classification behavior across different cancer types.

The Support Vector Machine (SVM) classifier with DenseNet-169 demonstrated notable stability, exhibiting the most consistent performance between training (84% accuracy, 0.98 AUC) and test sets (81% accuracy, 0.95 AUC). The ROC curves and confusion matrices for this combination are presented in Figs. (**3** and **4**), highlighting their robust discriminative capabilities across both datasets.

In contrast, the Artificial Neural Network (ANN) model with DenseNet-121 showed the lowest test set performance, achieving 81% accuracy on the training set and 73% accuracy (AUC 0.93) on the test set. Across all classifier types, DenseNet-169 and DenseNet-201 consistently outperformed DenseNet-121, with SVM models displaying more stable performance between training and test sets compared to ANN and MLP models. Table **1** provides a comprehensive summary of performance metrics for all model combinations.

Grad-CAM analysis was performed on 40 randomly selected sample images (10 per class) to evaluate model interpretability and decision-making patterns. The MLP model correctly predicted 36/40 samples (90.0% accuracy), while the SVM approximation model achieved 37/40 correct predictions (92.5% accuracy), on the visualization subset. Both models demonstrated focused attention on relevant anatomical regions and pathological features. Representative GradCAM examples from the best-performing model (MLP-DenseNet169, 83.0% test accuracy) are shown in Fig. (**5**) , illustrating the model's ability to identify clinically relevant regions across all lung cancer classes.

## 4 DISCUSSION

Building upon these results, the implications of these findings are examined in the context of current research and clinical applications. This study presents a systematic comparison of DenseNet architectures combined with different classifiers for lung cancer CT scan classification. The analysis extends beyond previous research by examining multiple classifier combinations, providing insights into their relative effectiveness for clinical applications.

The MLP-DenseNet-169 combination's achievement of 83% test accuracy represents a promising benchmark for automated lung cancer classification. This performance warrants comparison with recent literature in the field. Lanjewar *et al*. (2023) reported higher accuracies (95-100%) using a modified DenseNet-201 model with advanced feature selection methods. Their superior results likely stem from sophisticated feature selection techniques and architecture modifications specific to their dataset characteristics [9].

Recent developments in the field have shown various approaches to improving model performance:

### 4.1 Data Augmentation Approaches

Bumpenje *et al*. (2024) achieved comparable results (82.14%) using DenseNet-121 with enhanced data augmentation techniques, validating the current findings while highlighting the potential impact of advanced data preprocessing [16]. The SVM-based results align with Arzmi *et al*. (2023), who reported 87% accuracy with DenseNet-121-SVM, suggesting consistent performance patterns across different implementations [10].

### 4.2 Ensemble Methods

Recent developments by Quasar *et al*. (2024) demonstrated the potential of ensemble methods, achieving 98% accuracy through model integration. Their findings suggest that combining the best-performing models from the current study in an ensemble framework could enhance overall classification performance [17].

### 4.3 Interpretability Enhancements

Uddin *et al*. (2024) introduced an attention-enhanced DenseNet for non-small-cell lung cancer detection, presenting an innovative approach to improving model interpretability [8]. Additionally, Saied *et al*. (2023) provided a comprehensive comparison of AI methods for pulmonary nodule classification, reporting 90.39% accuracy with DenseNet-121 [7].

### 4.4 Efficiency *vs*. Resources

Our DenseNet-169 model achieved 83% accuracy on high-resolution CT scans, extending both the findings of Marappan *et al*. (2022), who demonstrated 76.67% accuracy using a lightweight 2D DenseNet on low-resolution CT scans [18], and Ciompi *et al*. (2017), who established DenseNet's effectiveness in pulmonary nodule management [19]. This 6.33% improvement while maintaining similar architectural principles suggests that our model effectively balances performance and resource requirements, validating key efficiency concepts in deep learning design [20-22].

### 4.5 Comparative Advantage

The current study distinguishes itself through its comprehensive evaluation of multiple classifiers across different DenseNet architectures. While some previous studies focused on optimizing single architectures or specific classifier combinations, this analysis provides insights into the comparative advantages of various model combinations, as summarized in Table **2**. The results demonstrate that DenseNet-169-based models offer an optimal balance between accuracy and generalization, particularly when combined with SVM or MLP classifiers.

## 5 LIMITATIONS AND FUTURE DIRECTIONS

Several limitations of the present study warrant discussion:

- The study lacks advanced feature selection and data augmentation techniques
- There is a focus on individual models rather than ensemble methods
- The study has a limited exploration of model interpretability
-Statistical significance testing between models was not performed due to the lack of systematic collection of individual sample-level predictions during the evaluation process.
-The current study utilized a dataset of 1,000 CT scan images from a single source (Kaggle), which may limit the generalizability of the findings across diverse patient populations and clinical settings.

Based on these limitations, future research will address:

1. Integration of advanced feature selection and data augmentation techniques
2. Exploration of ensemble methods to potentially enhance classification accuracy
3. Investigation of attention mechanisms or other interpretability-enhancing techniques
4. Development of lightweight model architectures for resource-constrained settings
5. Comprehensive comparison of model performance across various evaluation metrics and datasets
6. Implementation of k-fold cross-validation with variance reporting (confidence intervals) for more robust model evaluation.
7. Implementation of statistical significance testing (McNemar's test for classification accuracy and DeLong test for AUC comparison) to rigorously assess the significance of performance differences between models
8. Future studies should incorporate larger, multi-center datasets to improve model generalizability and accuracy. The integration of data from multiple medical institutions will provide more robust performance metrics and enhance the clinical applicability of deep learning approaches for lung cancer classification.

## CONCLUSION

This study demonstrates the effectiveness of combining DenseNet architectures with different classifiers for lung cancer CT image classification. The MLP-DenseNet-169 combination achieved optimal performance with 83% test accuracy, while SVM-DenseNet-169 showed superior stability with minimal performance degradation between training and test sets. The systematic comparison revealed that DenseNet-169 and DenseNet-201 consistently outperformed DenseNet-121 across all classifier types.

These findings provide valuable benchmarks for automated lung cancer detection systems and establish clear performance baselines for future research in medical image classification.

## Figures and Tables

**Fig. (1):**
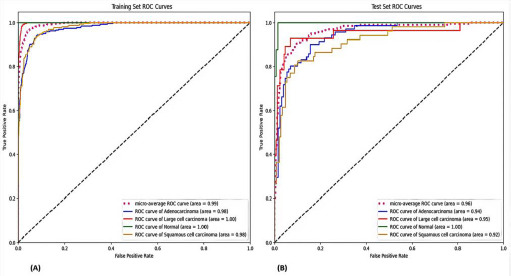
Receiver Operating Characteristic (ROC) curves for Multi-Layer Perceptron (MLP) classifier combined with DenseNet-169 architecture. (**A**) Training set performance showing AUC = 0.99 and 92% accuracy across four lung cancer classes (Adenocarcinoma, Large Cell Carcinoma, Squamous Cell Carcinoma, and Normal tissue). (**B**) Test set performance demonstrating AUC = 0.96 and 83% accuracy, indicating strong discriminative capability for lung cancer classification.

**Fig. (2):**
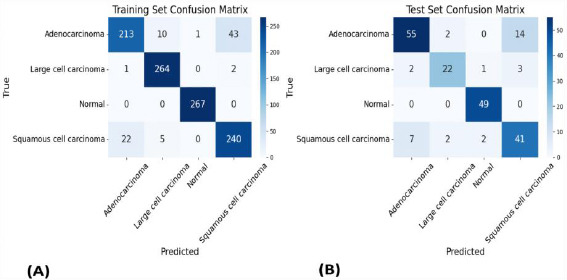
Confusion matrices for Multi-Layer Perceptron (MLP) classifier with DenseNet-169 showing classification performance across lung cancer subtypes. (**A**) Training set (**B**) Test set performance. Color intensity represents prediction confidence levels.

**Fig. (3):**
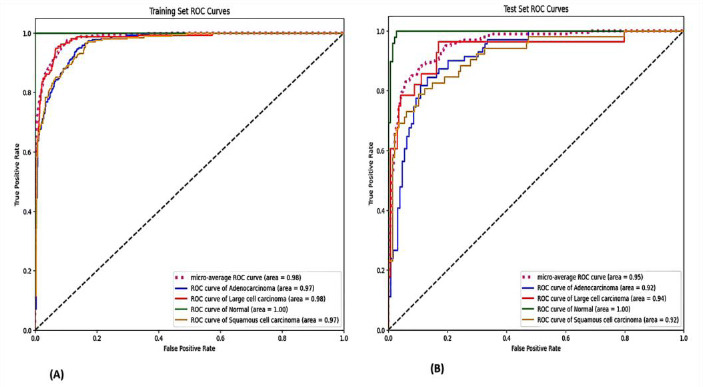
ROC curves for SVM-DenseNet169. (**A**) Training set: AUC = 0.98, 84% accuracy. (**B**) Test set: AUC = 0.95, 81% accuracy demonstrating stable generalization.

**Fig. (4):**
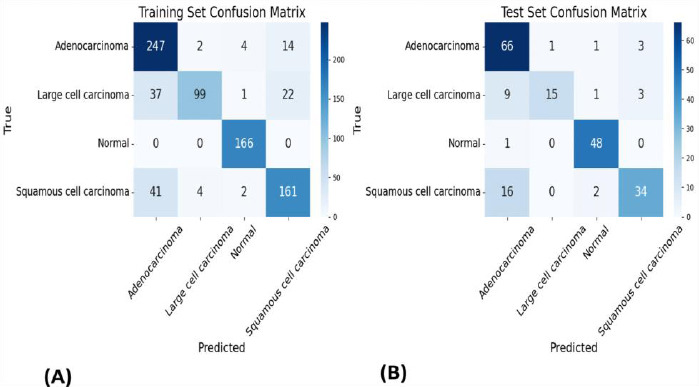
Confusion matrices for SVM-DenseNet169. (**A**) Training set and (**B**) Test set results showing balanced classification performance across all four lung cancer categories.

**Fig. (5):**
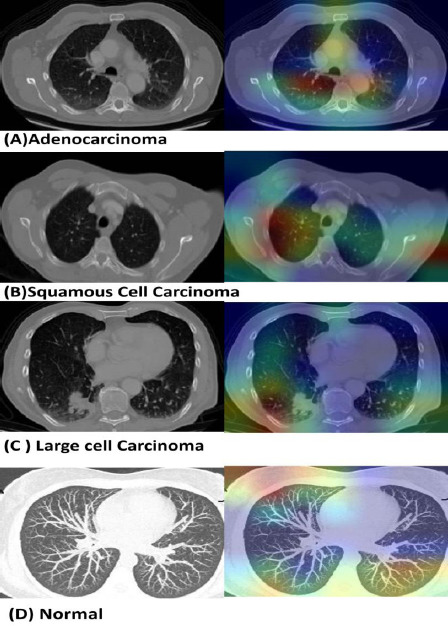
GradCAM visualization examples from MLP-DenseNet169 showing model attention regions for lung cancer classification. Left column: Original CT images. Right column: Superimposed GradCAM heatmaps highlighting regions contributing to classification decisions for (**A**) Adenocarcinoma, (**B**) Squamous cell carcinoma, (**C**) Large cell carcinoma, and (**D**) Normal tissue. Red/yellow regions indicate the highest model attention on pathologically relevant areas.

**Table 1:** Overall performance for training and test sets for all models included in this study.

**Model**	**Dataset**	**Accuracy**	**AUC**	**Recall**	**F1-Score**	**Precision**	**Specificity**
ANN201	Train	0.88	0.98	0.88	0.87	0.87	0.96
ANN201	Test	0.8	0.92	0.8	0.8	0.8	0.93
ANN169	Train	0.81	0.96	0.81	0.81	0.82	0.94
ANN169	Test	0.75	0.93	0.75	0.74	0.76	0.92
ANN121	Train	0.81	0.96	0.81	0.81	0.82	0.94
ANN121	Test	0.73	0.93	0.73	0.73	0.74	0.91
SVM201	Train	0.85	0.99	0.84	0.84	0.85	0.95
SVM201	Test	0.79	0.95	0.79	0.78	0.81	0.93
SVM169	Train	0.84	0.98	0.84	0.84	0.85	0.95
SVM169	Test	0.81	0.95	0.81	0.81	0.83	0.93
SVM121	Train	0.82	0.98	0.82	0.81	0.84	0.94
SVM121	Test	0.78	0.94	0.78	0.76	0.8	0.92
MLP201	Train	0.89	0.98	0.89	0.89	0.89	0.96
MLP201	Test	0.81	0.94	0.81	0.8	0.81	0.94
MLP169	Train	0.92	0.99	0.92	0.92	0.92	0.97
MLP169	Test	0.83	0.96	0.84	0.84	0.84	0.95
MLP121	Train	0.92	0.99	0.92	0.91	0.92	0.97
MLP121	Test	0.77	0.94	0.79	0.77	0.76	0.92

**Table 2:** A summarized comparison of the current study and previous studies.

**Study Reference**	**Methodology**	**Accuracy**	**Lung Conditions Classified**	**Significant Insights**
Current Study	DenseNet121, DenseNet169, DenseNet201 + SVM and ANN	82-88%	Adenocarcinoma, Large Cell Carcinoma, Squamous Cell Carcinoma, Normal	Use of multiple DenseNet architectures and three classifiers: SVM, ANN, and MLP, for comprehensive comparison
Bumpenje *et al*. (2024) [16]	DenseNet121 with albumentations and mixup data augmentation	82.14%	Adenocarcinoma, Large Cell Carcinoma, Squamous Cell Carcinoma, Normal	Combination of albumentations and mixup for data augmentation
Quasar *et al*. (2024) [17]	Ensemble model with BEiT, DenseNet, and Sequential CNN	98%	Not specified	Use of ensemble methods to improve detection accuracy
Uddin *et al*. (2024) [8]	ATT-DenseNet for CT and histopathological images	-	Non-Small-Cell Lung Cancer (NSCLC)	Integration of attention mechanisms with DenseNet for improved diagnostics
Lanjewar *et al*. (2023) [9]	Modified DenseNet201 with feature selection	95-100%	Adenocarcinoma, Large Cell Carcinoma, Squamous Cell Carcinoma, Normal	High accuracy using modified DenseNet201 and feature selection
Arzmi *et al*. (2023) [10]	DenseNet-SVM pipelines (DenseNet121-SVM, DenseNet169-SVM, DenseNet201-SVM)	87%	Normal, Large Cell Carcinoma, Adenocarcinoma, Squamous Cell Carcinoma	Evaluation of DenseNet-SVM combinations for classification
Saied *et al*. (2023) [7]	DenseNet-121, DenseNet-169, simple CNN, random forest, SVM on LIDC-IDRI dataset	90.39%	Pulmonary nodules	Comparison of deep learning and statistical methods
Marappan *et al*. (2022) [18]	Lightweight DenseNet for low-resolution CT images	-	Invasive Adenocarcinoma (IAC), Minimally Invasive Adenocarcinoma (MIA)	Use of low-resolution images with a lightweight model

## Data Availability

The data that support the findings of this study are available from the corresponding author, [Y.G], upon reasonable request.

## LIST OF ABBREVIATIONS

AI: Artificial Intelligence
ANN: Artificial Neural Network
API: Application Programming Interface
AUC: Area Under the Curve
CAD: Computer-aided Diagnosis
CNN: Convolutional Neural Network
CSV: Comma-Separated Values
CT: Computed Tomography
DenseNet: Densely Connected Convolutional Networks
GPU: Graphics Processing Unit
GradCAM: Gradient-weighted Class Activation Mapping
L2: L2 Regularization
MLP: Multi Layer Perceptron
RAM: Random Access Memory
RBF: Radial Basis Function
ReLU: Rectified Linear Unit
ROC: Receiver Operating Characteristic
SVM: Support Vector Machine
